# Infectious Aetiology of Marginal Zone Lymphoma and Role of Anti-Infective Therapy

**DOI:** 10.4084/MJHID.2016.006

**Published:** 2016-01-01

**Authors:** Salvatore Perrone, Gianna Maria D’Elia, Giorgia Annechini, Alessandro Pulsoni

**Affiliations:** Division of Hematology, Department of Cellular Biotechnologies and Hematology, “Sapienza University” Rome, Italy.

## Abstract

Marginal zone lymphomas have been associated with several infectious agents covering both viral and bacterial pathogens and in some cases a clear aetiological role has been established. Pathogenetic mechanisms are currently not completely understood. However, the role of chronic stimulation of the host immune response with persistent lymphocyte activation represents the most convincing explanation for lymphoproliferation. Gastric MALT lymphoma is strictly associated with *Helicobacter pylori* infection and various eradicating protocols, developed due to increasing antibiotic resistance, represent the first line therapy for gastric MALT. The response rate to eradication is good with 80% of response at 1 year; this finding is also noteworthy because it recapitulates cancer cured only by the antibacterial approach and it satisfies the Koch postulates of causation, establishing a causative relationship between Hp and gastric MALT lymphoma. Patients with chronic HCV infection have 5 times higher risk to develop MZL, in particular, an association with splenic and nodal MZL has been shown in several studies. Moreover, there is evidence of lymphoma regression after antiviral therapy with interferon+ribavirin, thus raising hope that newly available drugs, extremely efficient against HCV replication, could improve outcome also in HCV-driven lymphomas. Another case-study are represented by those rare cases of MZL localized to orbital fat and eye conjunctivas that have been associated with *Chlamydophila psittaci* infection carried by birds. Efficacy of antibacterial therapy against C. psittaci are conflicting and generally poorer than gastric MALT. Finally, some case reports will cover the relationship between primary cutaneous B-cell Lymphomas and *Borrelia Burgdorferi*.

## Introduction

Marginal zone lymphoma (MZL) is a heterogeneous group of low-grade, B-cell, non-Hodgkin lymphomas comprising three distinct diseases: extranodal MZL (EMZL) of mucosa-associated lymphoid tissue (MALT), splenic MZL (SMZL), and nodal MZL (NMZL).^1^ In recent European studies, MZL has been reported in 12% of new lymphoma diagnoses,^2^ while a lower incidence of 3% was reported in the United States from Surveillance, Epidemiology, and End Results (SEER) registries.^3^ Various contributing factors have been defined in terms of pathogenesis, including autoimmune diseases (mainly Sjögren syndrome and chronic autoimmune thyroiditis)^4^ while also several infectious agents are known or simply suspected to cause MZL. Mainly three mechanisms linking infections to MZL have been hypothesized:

1. lymphocyte-transforming viruses can infect lymphocytes inducing dysregulation of normal cell functions and promotion of cell division (Epstein–Barr virus, Human Herpesvirus 8, Human T-lymphotropic virus type I);
2. induction of immune suppression through progressive depletion of CD4+ T-lymphocytes, as in AIDS (Human immunodeficiency virus) or in patients submitted to therapeutic immunosuppression;
3. stimulation of chronic immune response and persistent lymphocyte activation (Helicobacter pylori, Hepatitis C virus, *Chlamydophila psittaci*, *Borrelia burgdorferi*, *Campylobacter jejuni*).^5^

Specific organisms have been implicated in the aetiology of MZL involving particular anatomic sites:^6^ *Helicobacter pylori* for the stomach, *Chlamydophila psittaci* for the ocular adnexa, *Borrelia burgdorferi* for the cutis, *Campylobacter jejune* for the small intestine, *Mycobacterium spp.* For the bronchus, Hepatitis C virus (HCV) for splenic and nodal MZL. However, a robust association is present only in gastric MALT lymphoma with *Helicobacter pylori (Hp*), where 90% of cases are Hp+.^7^ Moreover, Hp eradication therapy is effective in gastric MALT Hp+ lymphoma with a remission rate around 80%^8^,^9^ and also long-term results are excellent.^10^ The studies on *Hp* and gastric MALT lymphoma have profoundly influenced our understanding of the pathogenesis of lymphomas and modified our management, because, for the first time in the history of medical oncology, cancer has been cured by antibiotic therapy.

This review focuses on the role of pathogens and development of MZL, with implication on the therapeutic option to target the implied infectious agents.

## Gastric MALT Lymphoma

The clinical presentation of gastric involvement by MALT lymphoma is variable and not specific with abdominal pain being the most common symptom, followed by dyspepsia, vomiting, nausea and anorexia; weight loss is common; gastric bleeding occurs as presenting symptom in 20%–30% of patients, while gastric occlusion and perforation are less common.^11^ Diagnosis is made after histopathological evaluation of gastric biopsy, generally after esophagogastroduodenoscopy, and rely on the morphologic demonstration of the “hallmark” of MALT lymphoma: the lymphoepithelial lesion that results from invasion by atypical lymphocytes of epithelial mucosa and invasion of the glandular epithelium, as well as reactive lymphoid follicles.^12^ Paradoxically, the stomach is the commonest site of MALT lymphoma, despite MALT tissue is not normally present in gastric mucosa. However, differentiation from other indolent lymphomas (Follicular lymphoma, Mantle-cell lymphoma) and aggressive lymphoma (Diffuse large B-cell lymphoma and Peripheral T-cell lymphoma) is not always straightforward, and a minimum immunohistochemistry panel including CD20, CD10, CD5 and cyclin D1 is recommended.^13^ Since the diagnosis should be made according to the WHO criteria,^14^ it should be reviewed by an expert hematopathologist.^15^ Since the first discovery in 1982 by the Nobel prize-winning authors, B. Marshall and R. Warren^16^ *Helicobacter pylori* has become critical for treating and studying gastric disease like peptic ulcer, MALT lymphoma and gastric cancer.^17^ Hp identification mainly relies on histology (HE and modified Giemsa staining) and culture or invasive molecular tests.^18^ In the case of negativity, serology should be performed to identify truly negative gastric MALT lymphomas,^19^ in fact, Hp mucosal colonisation is not uniform, but in patches;^20^ therefore, the infection can go undiagnosed if biopsy involves a non-colonised area. In addition, it is believed that extensive mucosal lesion secondary to lymphoma may reduce the density of the infection to even undetectable levels.^21^,^22^

### Role of *H. Pylori* in MALT Lymphoma

Helicobacter species are the indigenous biota of mammalian stomachs, and *H. pylori* is the human-specific inhabitant,^23^ there is evidence that *H. pylori* has been present in humans at least since ancestors of Amerindians migrated from Asia more than 11,000 years ago.^24^ In this long time span, Hp has adapted to human gastric environment establishing an interaction that can be interpreted as both commensalism and long-term parasitism.^25^ Extensive allelic diversity and genetic variability are hallmarks of this microaerophilic gram-negative bacterium,^26^ resulting from the combination of a high mutation rate^27^ and frequent exchange of genetic material during mixed strains infections.^28^ That extraordinary capacity of adaptation to human host (resembling a quasispecies) is the key to the success for this microorganism to infect more than one-half of the human population. *H. pylori* has been established by *International Agency for Research on Cancer* (IARC) to be definitive bacterial carcinogen for humans^29^ and is estimated to be responsible for 5.5% of all human cancer cases, about 592.000 new gastric cancer.^30^ Spectrum of H. pylori infection is wide with most carriers remaining asymptomatic while patients with duodenal ulcer have antral predominant gastritis with little mucosal atrophy and hyperacidity; patients with gastric ulcer almost invariably have corpus predominant gastritis and hypoacidity with various degree of mucosal atrophy,^31^ the latter condition is associated with gastric cancer.^32^ Moreover, the case of gastric MALT lymphoma is a rarer condition, accounting for <5% of primary gastric neoplasms. The epidemiological data raise the question why some people develop gastric cancer (also MALT lymphoma), and others do not? For example, what is the possible answer to the ‘African enigma’ where ubiquitous *H. pylori* infections is not associated with gastric cancer but only gastritis?^33^ On the other hand, in the setting of a developed Country like the Nord-East of Italy, where there is a higher prevalence of HP infection there is a concomitant high incidence of gastric lymphoma.^34^ Given that some simulations indicate that Hp seems to have spread from East Africa around 58,000 years ago following human migrations,^35^ we could hypothesize that in Africa, where *H. pylori* have interacted for more time with its human host, it could have reached a better symbiotic state with a decreased virulence.

### Pathogenesis of *Helicobacter Pylori*-Induced Gastric MALT Lymphoma

There are several known mechanism that *Helicobacter* exploits to interact with the host mucosa. Actually, *H. pylori* strains can be broadly categorized into strains that express multiple factors that interact with host tissue (CagA, s1-VacA, BabA, OipA) and strains that lack these factors (36). In 1989, CagA was firstly identified 37 and is now recognized as a marker for strains that confers increased risk for peptic ulcer disease^38^ and gastric cancer.^39^ The Cag Type IV secretion system (T4SS) is the primary virulence determinant and is responsible for injecting macromolecules, in particular, CagA, inside epithelial cells.^40^ CagA is then tyrosine-phosphorylated by the host cell Src kinase; once phosphorylated, CagA interacts with SHP-2, a tyrosine phosphatase, which affects spreading, migration, and adhesion of epithelial cells.^41^ Moreover, CagA protein interacts with Grb2 and activates the Ras/MEK/ERK pathway, leading to the phenotypes of cell scattering and proliferation.^42^ In addition, tyrosine-phosphorylated CagA binds and activates C-terminal Src kinase (Csk) via its SH2 domain, which in turn inactivates the Src family of protein-tyrosine kinases. Since this signalling may induce apoptosis, the Csk pathway may attenuate the other CagA interactions.^43^ In conclusion, attenuation of CagA activity by Csk may enable CagA-positive *HP* to infect the human stomach persistently for decades while avoiding excessive CagA toxicity to the host.^43^ There is also evidence that CagA can be directly injected by T4SS inside B-lymphocytes. The delivered CagA induces the activation and stimulation of B cells (mediated by intracellular SHP-2 and phosphorylation of ERK and P38 MAPK), and could initiate the first step of transformation, also upregulating anti-apoptotic proteins BCl-2 and BCl-XL.^44^ Moreover, Kuo and colleagues have demonstrated the presence of the bacterial protein CagA inside malignant B cells of MALT lymphoma and that those patients tend to respond faster to *HP* eradication.^45^

The vacuolating cytotoxin VacA, a high–molecular weight multimeric pore-forming protein is one of virulence factors produced by Hp and is responsible for epithelial cells by both apoptosis and programmed cell necrosis.^46^ VacA forms a pore that permeabilizes the epithelial cell plasma membrane to urea,^47^ that is an essential substrate for the *H. pylori*’s urease to mitigate acid gastric environment. More importantly, VacA determines immune suppression by blocking phagosome maturation in macrophages,^48^ inhibiting antigen presentation in T cells,^49^ blocking T cell proliferation with down-regulation of Th1 effects through interaction with calcineurin to block intracellular signalling.^50^

Chronic *H. pylori* infection can trigger inflammation and immunological responses that stimulate lymphoid infiltration displaying features of classic MALT architecture.^51^ It is suggested that *H. pylori* infection results in T-cell-dependent responses through the classic germinal centre reaction, with generation of reactive B and T cells; the H. pylori-specific T cells then migrate to the marginal zone/tumour area and provide non-cognate help to auto-reactive neoplastic B cells, which may involve stimulation of CD40 and other surface receptors by soluble ligands and cytokines.^52^,^53^

Host polymorphisms are also a crucial determinant of the interaction with *H. pylori* and could elucidate why only rarely MALT lymphoma develops. Determinants of host immune response variability have been extensively reviewed by Datta De^36^ and mainly consist in variation in an inflammatory gene like IL-1 (*RN 2/2* genotype), glutathione S-transferase T1 null phenotype^54^ and a negative association with HLA-B35.^55^

An important proof of concept of the association of Hp with Gastric MALT were the first report by Wotherspoon of MALT regression after successful eradication of Hp.^56^ Those data have been corroborated by other studies,^8^,^10^ thus fulfilling Hill’s criteria of causality.

### Gastric MALT Beyond *Helicobacter Pylori.*

After a clonal expansion of B-cell *H. pylori*-driven has established in gastric mucosa, other mutational events can explain the gain of independence from the infection itself. Three chromosomal translocations are the most frequently detected t(11;18)(q21;q21)/API2-MALT1, t(1;14)(p22;q32)/BCL10-IGH, and t(14;18)(q32;q21)/IGH-MALT1, all converging to activation of nuclear factor kappa-B (NF-κB).^53^ Translocation t(11;18)(q21;q21), occurring in approximately 30% of cases, fuses the N-terminus of the API2 gene to the C-terminus of the MALT1 gene and generates a functional API2-MALT1 fusion product,^57^,^58^ the other two translocations involve translocation to immunoglobulin gene loci, and consequently increased expression, of BCL10 and MALT1 genes.^12^ Gastric MALT lymphomas carrying the t(11;18) are more aggressive with spread to local lymph nodes 59 and, most importantly, rarely respond to HP eradication.^60^,^61^ Therefore, its detection can influence the clinical management and is easily feasible with a commercial MALT1 dual-colour break-apart probe and an API2-MALT1 dual-colour dual-fusion probe for FISH, or by rt-PCR of the API2-MALT1 fusion mRNA transcripts.^62^ Not translocated cases frequently carry trisomies of chromosomes 3, 12 and 18.^63^

### Gastric MALT lymphoma *H. Pylori* Negative

Another interesting setting is represented by gastric MALT lymphoma, *H. pylori* negative that responded to antibiotic treatment in 15.5% of cases, despite *H. pylori* triple negativity test.^64^,^65^ Those data have been interpreted by authors in light of a limited accuracy of *H. pylori* detection (false negative) or the possible presence of other unidentified bacteria implicated in MALT pathogenesis.^65^ Nowadays, given the limited toxicity, low costs and risk of low-grade lymphoma progression, antibiotic therapy is also recommended in *H. pylori*- cases.^66^

### Antibiotic Treatment

As previously discussed, targeting *H. pylori* seems a logical first line approach for gastric MALT lymphoma. Several effective schemes are available for the treatment of *H pylori* infection.^67^–^69^ The antibiotic choice should be based mainly on the epidemiology of Hp resistance to clarithromycin. Therefore, in countries with a prevalence >15% (That is the case of Europe, with the exception of Northern States)^70^ antimicrobial susceptibility testing could be useful.^18^ The most commonly used regimen is triple therapy: a proton pump inhibitor (omeprazole)^71^ in association with amoxicillin and clarithromycin.^72^ Metronidazole can be substituted to amoxicillin in penicillin-allergic individuals. An alternative is represented by the Sequential treatment, which includes a 5-day period with PPI+amoxicillin, followed by a 5-day period with PPI-clarithromycin-metronidazole.^73^ For failure of the first line therapy or for clarithromycin-resistant isolated strains, it is available a bismuth-based quadruple therapy with omeprazole, bismuth salts, metronidazole and tetracycline (OBMT),^74^ an RCT using a combination of PPI and a single three-in-one capsule tablet showed improved eradication in comparison with standard triple therapy.^75^ Finally, in case of failure of the second-line treatment (bismuth-containing quadruple regimen), it is recommended to use the PPI-levofloxacin-amoxicillin regimen,^76^,^77^ always considering the rise in epidemiologic *H. pylori* resistance to levofloxacin.^68^

MALT lymphoma response to *H. pylori* eradication is about 80%.^65^ However the length of time necessary to obtain a remission can span from few months to more than 12 months.

### Anti-Lymphoma Directed Treatment

In cases not responding to antibiotic treatment, a control of localised disease can be achieved with radiation therapy alone with moderate-dose involved-field radiotherapy (24–30 Gy) to the stomach and perigastric nodes.^13^,^78^

Patients with the symptomatic systemic disease should be considered for systemic treatment that encompass the association of rituximab + chemotherapy. Rituximab in combination with chlorambucil has been proven in a randomised study by International Extranodal Lymphoma Study Group (IELSG)-19 where an excellent event-free-survival was achieved, superior to chlorambucil alone, although no overall survival benefit has been shown. Aggressive anthracycline-containing regimens (CHOP-like) are not usually necessary and should be reserved for cases with transformation to high grade.^13^

## Marginal Zone Lymphoma and HCV

A heterogeneous group of lymphoproliferative disorders have been long suspected to be associated with HCV infection. HCV-related Mixed cryoglobulinemia (MC) is considered as a low-grade B-cell lymphoproliferative disorder, characterized clinically by arthritis, cutaneous vasculitis (palpable purpura), and, occasionally associated with glomerulonephritis and by the presence of circulating cryoprecipitable immune complexes of more than one immunoglobulin class. MC is defined by laboratory findings: the presence of serum Ig that precipitate at low temperatures (< 37°C) and can solve by warming serum, that are produced by the lymphoproliferation of B-cells clones secreting pathogenic IgM with rheumatoid factor activity. MC can evolve into an overt B-cell NHL in approximately 8–10% of cases after a long period.^79^

MZLs, in particular splenic (SMZL) and nodal MZL (NMZL), and other extranodal-MZL are the iNHL subtypes most frequently described as being HCV-related (80–82). SMZL is an indolent and rare entity, separately recognised by WHO,^1^,^83^ usually presenting with symptomatic splenomegaly, cytopenias, autoimmune phenomena, and serum monoclonal paraprotein; in some patients a leukemic phase characterized by circulating lymphocytes with villous projections defines the so-called Splenic lymphoma with villous lymphocytes (SLVL).^84^ SMZL, albeit rare, in a population-based study, has been reported with an incidence between 2001 and 2008 of 0.13 per 100,000 person-years, accounting for 0.6% of all NHL cases^85^ and is the most common primary splenic lymphoma.^86^ SMZL incidence is higher among older (median age is 65 years), white and male (male-to-female ratio of 1.2:1) population, in the United States.^85^

NMZL is a distinct clinical-pathological subtype of MZL characterized by exclusive primary lymph node localization in the absence of extranodal or splenic disease.^1^ NMZLs represent only 1.5% to 1.8% of all lymphoid neoplasms; the most frequent clinical presentation is a generalized or, less frequently, localized lymphadenopathy.

Recently, Paulli et al. have reported a new subset of patients with extranodal HCV-related MZL characterized by a primary ‘lipoma-like’ subcutaneous presentation and an indolent clinical course.^87^

### Role of HCV in iNHL

Hepatitis C virus (HCV) is an enveloped, RNA virus of the Flaviviridae family; it comprises six major genotypes, whose prevalence varies among different population and countries. HCV is not only hepatotropic, causing infection of hepatocytes associated with hepatitis, liver cirrhosis and hepatocellular carcinoma (HCC), but is also responsible for other extrahepatic manifestations, of whose the most frequent is lymphoproliferation.^88^

### Models of Pathogenesis in HCV-Induced Lymphoproliferation

The mechanisms underlying B-cell lymphoproliferation possibly induced, directly or indirectly, by HCV chronic infection are yet not fully understood. However, few experimental data suggest some clues to explain this phenomenon and have been reviewed elsewhere.^89^,^90^

The first observation derives from the HCV chronic antigenic stimulation leading to continuous stimulation and selection of reactive B-clones. HCV-associated NHLs derive from B cells activated during HCV infection, with some of these B cells being specific for the protein HCV-E2, which is the primary target of antibody responses against HCV.^91^ Moreover, the monoclonal IgM component of type II MC is often encoded by the same set of Variable region genes, V_H_1–69, and V_k_3-A27 and similarly the same genes can be demonstrated to be involved in some cases of HCV-related NHL.^92^,^93^ The burden of these data suggests that HCV-associated lymphomas are derived from clonally expanded B cells stimulated by HCV-E2 protein.

HCV is a positive, single-strand RNA virus, lacking a DNA intermediate in its replicative cycle. Thus, insertional mutagenesis in infected B-cells seems not possible. Moreover, there is conflicting evidence about the demonstration in B-cells of the negative RNA viral strand, which would be consistent with active viral replication. In fact, in a Japanese study negative-strand HCV RNA was detected in B cells only in 4 of the 75 (5%) patients^94^ while no replicative intermediates were detected in another study.^95^ In addition, B-cells could be not suitable for virus entry because claudin-1, a membrane co-receptor required for HCV infection is not present in peripheral blood cells.^96^

Conversely, more robust data exist supporting a direct B-cell stimulation by the engagement of CD81 on their surface by a combination of HCV-E2 and anti-CD81 antibodies leading to polyclonal activation of naïve, CD27- B-cells.^97^

### Antiviral Therapy

Since 1990, several Epidemiologic studies suggested an association between hepatitis C virus (HCV) infection and B-cell NHL, although with geographical heterogeneity due to the different prevalence of HCV seropositivity.^98^–^102^ Nevertheless, many of these studies suffered from methodologic restrictions such as their retrospective nature, the consideration of prevalent instead of incident cases, missing or inappropriate controls. In 2003, the GIMEMA group published an Italian case-control study performed with adequate epidemiologic methods that demonstrated a clear association between HCV infection and various types of B-NHL.^103^ Recently a larger case-control study of InterLymph NHL Subtypes Project hepatitis confirmed the increased risk of MZL in C virus seropositive patients (EMZL OR = 5.29, 95% CI = 2.48 to 11.28).^104^ In 2002, Hermine et al. reported the first series of nine patients with SLVL in which a complete regression of lymphoma was obtained with antiviral treatment (AT) consisting of interferon (IFN) +/− ribavirin.^105^ As a control, the authors reported 6 pts. with the same disease, but without HCV infection, not responding to the same treatment. This argument suggested for the first time that viral eradication and not the direct anti-proliferative effect of IFN was responsible for HCV-related NHL regression. Many single cases subsequently confirmed this observation, although, due to the rarity of HCV-related NHL, very few prospective studies have been published. In 20 patients, Vallisa et al. demonstrated a complete response to antiviral treatment of different subtypes in 70% of indolent i-NHL.^106^ Recently a multicenter study of Fondazione Italiana Linfomi (FIL) recorded more than 700 patients with i-NHL and HCV seropositivity, demonstrating that AT used in the first line in 100 pts produced 44 CR and 33 PR.^107^ A French prospective study of 116 HCV-positive patients with B-NHL revealed that within the MZL subgroup (n=45 pts.), 84% (n=38) received AT and 61% (n=23) achieved a Sustained Virological Response.^108^ Moreover, outcome analysis showed a favourable association between OS and AT in MZL patients (P = 0.04).^108^ From these experiences, AT is, to date, considered the first-line approach for the cases of HCV-related indolent lymphoma, not requiring immediate chemo-immunotherapy. The recent introduction of new, highly active, antiviral treatments could clarify whether a pure antiviral approach,^109^–^111^ free from the confounding role of interferon,^112^ is equally or even more efficacious against lymphoma.

## Ocular Adnexa MALT Lymphoma

Ocular adnexa MALT lymphoma (OAML), although uncommon, is the third most frequently involved site of MZL with an incidence rate of 1.4/1,000,000 person-years, with a median age of 65 years,^6^ thus accounting for 50–78% of all ocular lymphomas in Western Countries.^113^ Moreover, Danish and American SEER data from surveillance registries have reported an increase in the incidence of OAML.^114^,^115^ Clinical presentation of orbital fat lymphomas involvement (75% of OAML) includes exophthalmos (27% of cases), palpable mass (19%), eyelid ptosis (6%), diplopia (2%), eyelid nodule, orbital oedema, epiphora and a variable degree of impaired ocular motility, while the most common sign for conjunctival lymphomas (25% of OAML) is the characteristic ‘salmon red patch’.^116^ OAML is an indolent lymphoma with a favourable clinical outcome emphasized by a 10-year overall survival of 81%, with no deaths from lymphoma for the patients treated with radiotherapy 117 and 94% for the patients managed with ‘watch and wait’.^118^ Microscopically neoplastic cells are monocytoid, centrocytic-like or lymphoplasmocitoid and their immunophenotype is similar to that of other MALT lymphomas: CD20+, CD79a+, usually IgM+ with light-chain restriction, PAX5+, bcl-2+, TCL1+, CD11c+/−, CD43+/−, CD21+/−, CD35+/−, and IgD2, CD32, CD52, CD102, CD23-, cyclin D1-, bcl-6-, MUM1-.^119^ Frequent chromosomal alteration encompasses trisomy 3 in 62% of cases and trisomy 18 in 47% of cases.^120^ In addition, the most common translocations in ocular adnexal MALT lymphomas are the t(11;18)(q21;q21)/API2-MALT1 and the t(14;18) (q32;q21)/IGH-MALT1.^121^ The immunoglobulin heavy-chain gene rearrangement is clonal in 55% of cases^122^ and shows somatic hypermutation in two-thirds of these,^123^ with a majority of selected genes commonly implicated in the assembly of autoantibodies, hence supporting the view that OAML represents a clonal expansion of post-germinal-centre memory B-cells, where selection may have occurred driven by antigen stimulation.^123^ Moreover, in OAML a possible infectious association has been long proposed, *Chlamidophila spp.* and, to a lesser extent, H. pylori and HCV^124^ have been proposed as causative agents. Although the association with Hp is controversial,^125^ a more robust evidence is available for the role of *C. psittaci*.^119^,^126^

### Role of *C. Psittaci* in Ocular Adnexa MALT Lymphoma

*Chlamydophila psittaci* (CP) is an obligate intracellular bacterium responsible for psittacosis/ornithosis in birds and in humans after zoonotic infection through inhalation of aerosolized bacteria when exposed to infected birds or handling contaminated feathers, faecal material or carcasses.^127^ CP infection is commonly asymptomatic with repeated infection cycles in humans but mainly involves the respiratory tract. CP has been reported as a potential trigger for OAML, and Ferreri et colleagues firstly showed the efficacy of antibiotic treatment.^126^ In this work CP DNA was found in lymphoma samples from 32 of the 40 (80%) case-patients analysed, thus revealing a strong association between OAML and CP infection, also in light of the low seroprevalence in general population studies varying between 0 and 49% (median: 5–10%).^128^ Moreover, Ponzoni et al. reported the presence of CP in 74% OAML specimens by different techniques such as immunohistochemistry, immunofluorescence and laser-capture microdissection-assisted PCR inside infiltrating monocytes/macrophages.^129^ Nevertheless, significant variability in CP association with OAML has been reported in different geographical areas, ranging from 47% in Germany to 35% in the East Coast of the USA, 29% in the Netherlands, 13% in Italy, 12% in UK and 11% in Southern China,^130^ while no evidence of CP infection was found in cases from the South Florida 131 and Japan.^132^ To sum up, the overall prevalence of *CP* infection in 423 cases of OAML reported in 14 different papers is 19%.^128^ In conclusion, the possible role of methodological pitfalls and other interpretation bias or confounding factors should be carefully considered when interpreting the bacteria–lymphoma association, also focussing on the potential role for *C. psittaci* infection in lymphomagenesis.^128^

### Treatment

OAML is a rare indolent lymphoma, for its treatment no consensus is available because no prospective clinical trials have been conducted to define the optimal treatment approach for these patients.^133^ However, patients managed by a watch & wait for approach have a 10-years OS of 94%.^118^ Moreover, limited toxicity and costs associated with antibiotic treatment should suggest the opportunity to target the possibly correlated infection by *C. psittaci,* in analogy with gastric MALT lymphoma and Hp eradication. A single antibiotic course of oral doxycycline at a dose of 100 mg, given twice a day, for 3 weeks is the most popular regimen. In the first prospective trial of doxycycline, 20 of the 27 patients were progression free at 2 years; interestingly also 6 CP DNA–negative patients of the 16 treated experienced lymphoma regression.^134^ A subsequent international prospective phase II trial was performed by IELSG: the prevalence of Cp positivity in OAML was 89%; in these naïve patients, after CP eradication, lymphoma regressed with an ORR of 65%, with 6 complete and 16 partial responses.^135^ A larger Korean prospective trial, enrolling 90 patients, showed an ORR of 27%.^136^ Actually, a new prospective study (IELSG 39) is enrolling patients (http://www.ielsg.org/trialsonfr.html). On the contrary, another retrospective study did not show any response,^137^ even if the short median follow-up of 9 months could have hampered the proper assessment of response. Altogether, in 9 studies identified in the literature by Kiesewetter and Raderer, 131 patients were treated with doxycycline resulting in an ORR of 45%, with CR achieved in 23 patients (18%) and PR achieved by 36 pts. (27%) (138). An interesting Italian work by Govi et al. showed the efficacy of administration of 500 mg clarithromycin, twice a day, for 6-months, in relapsed/refractory EMZL after treatment with doxycycline.^139^ Over the anti-bacterial effect on unidentified pathogens, clarithromycin could exert a direct anti-proliferating effect on OAML. Patients who fail to respond to doxycycline therapy can be successfully salvaged with chemotherapy and/or radiotherapy.

### Anti-Lymphoma Directed Treatment

Standard treatment is based on surgical resection of the single lesion. Radiotherapy is known as a treatment modality with a high local control rate for primary OAML.^140^,^141^ However, orbital irradiation can induce complications such as cataracts, keratitis, dry eye syndrome, and retinopathy.^142^ Even if no universally accepted radiation schedule is available, National Cancer Center Network guidelines recommend radiotherapy of 20 to 30 Gy for initial treatment of early-stage non-gastric MZL of all sites and reirradiation for locally recurrent disease.

Only limited data on chemotherapy for patients with OAML suggest different association and schedule. The oral agent chlorambucil is the most frequently used chemotherapy agent and has an extremely favourable toxicity. Complete responses are observed in 67% to 100% of patients; however, long-term outcome data suggest that local recurrence occurs in up to 29% of patients.^143^–^146^ Only a few cases of OAML have been treated with rituximab as single-agent, demonstrating high activity in both newly diagnosed and relapsed disease, although early recurrence is common.^147^–^150^ Also, the association of rituximab and chlorambucil has been tested with encouraging results.^151^ CNS prophylaxis is not recommended since OAML rarely recurs to CNS.^152^

## Role of Borrelia Burgdorferi in Primary Cutaneous B-cell Lymphomas

MZL of the skin has an incidence rate of 1.1/1,000,000 person-years 6 and is predominant among males across all ages. *B. burgdorferi (Bb)* infection has been associated with skin MZL in some cases in Europe, but not in the U.S., Asia and some parts of Europe, thereby challenging the aetiological role of this agent.^153^,^154^ In particular, in Bb endemic areas such as the Scottish Highlands^155^ or Austria,^156^ cutaneous MZL patients have demonstrated Borrelia infection in up to 40% cases, while no association was detected in two Italian case series.^157^,^158^ However, in a nonendemic region like France, Bb DNA was found in 19% of 16 cases with primary cutaneous MALT lymphoma.^159^

Bb infection might be associated with chronic antigen-driven lymphomagenesis in the skin, which is the port of entry of this gram-negative spirochete, through a bite from Ixodid tick and is also the infectious agent of Lyme borreliosis.^5^,^160^ Moreover, in late Lyme borreliosis, lymphocytes may infiltrate the dermis and produce the characteristic borrelia “lymphocytoma”, a cutaneous B-cell pseudolymphoma characterized by ‘top-heavy’, mixed-cell lymphoid infiltrate, usually accompanied by the formation of lymphoid follicles with germinal centres.^161^ Lyme disease and primary cutaneous lymphoma may represent a continuous spectrum of pathological states viewed as a multistep progression from lymphocytoma to “pseudolymphoma” eventually leading to primary cutaneous B-cell Lymphoma, where evidence of B-cell monoclonality may help distinguish between the different stages of the disease.^5^

Discordant data exist about cutaneous MZL recession after antibiotic treatment of Bb infection (generally consisting of cephalosporins +/− tetracyclines) and are based on case reports.^138^ In conclusion, Bb and its association with cutaneous MZL are currently the object of investigation, even if an antibiotic treatment may be attempted given the indolent nature of the disease.^162^

## Concluding Remarks

Marginal zone lymphoma is a fascinating clinical setting in which it has been clearly shown in several trials (see Table 1) that several therapies targeting the putative oncogenic infectious agent can induce steady lymphoma regression. It represents the proof of concept that a chronic stimulus on the immune system induced by an infectious agent, under particular host predisposition, may lead to the selection of abnormal B-clones and a more selection may result in overt lymphoma development. For gastric and the ocular adnexa MALT lymphomas the compelling evidence, to date, provides a rationale to implement actively antibiotics regimens that can be an effective first-line treatment due to the peculiar indolent course of the disease and the high therapeutic index of these drugs. Indeed, international guidelines state that “Helicobacter pylori eradication therapy must be given to all gastric MALT lymphomas, independently of stage”. Conversely, in OAML and MZL of the skin antibiotic treatment remains investigational, given the questionable results of the former and the paucity of data for the latter clinical setting.

With the recent availability of new direct-acting antiviral agents for HCV infection, that promise a rapid and sustained virological response, there is increasing interest in their employment to treat HCV-related SMZL and NMZL; however, further studies are needed to assess this strategy. In conclusion, we hope new evidence can improve our understanding of the pathogenesis of lymphoma mediated by antigen-dependent infectious agents, thus enabling the availability of other alternative and efficacious anti-lymphoma treatments.

## Figures and Tables

**Table 1:** Most relevant studies evaluating the efficacy of anti-infectious therapy for MZL regression.

Reference	Disease	Year	Patients	Antibacterial/Antiviral therapy	ORR	Response	Median follow-up (range) *in months*
Wundish T^163^	Gastric MALT	2005	120	*First line:* amoxicillin + omeprazole for 2 weeks *Second line*:metronidazole + omeprazole + clarithromycin for 10 days	90%	CR 96 (80%) PR 13 (10%) SD 11 (9%)	75 (2–116)
Kim JS^164^	Gastric MALT	2007	111	*First line:* omeprazole + amoxicillin + clarithromycin *Second line:* omeprazole + bismuthate + metronidazole + tetracycline	85%	CR 84 out of 99 cases (84.8%) SD 15 out of 99 cases (15.2%)	41 (11–125)
Raderer M^165^	Gastric MALT *H pylori* Negative	2015	24 (13)	Clarithromycin in all patients, +/− metronidazole+/− amoxicillin +PPI	46%	CR 5 (38%) PR 1 ( 7%) SD 4 (31%) PD 3 (23%)	95 (42–181)
Arcaini L^107^	SMZL 27% MALT 22%	2014	134	IFN (47) (IFN+RBV 36) peg-IFN 87 (IFN+RBV in 82)	77%	*First line:* CR 44 (44%) PR 33(33%) SD 14(14%)	NA
Michot JM^108^	MZL 39% DLBCL 39%	2015	45	Peg-interferon α + ribavirin In 6 pts peg-IFN+RBN+protease inhibitor	AT 78% AT-R 100%	AT only 14pts CR 8 PR 3 AT+R 8 pts CR 3 PR 5	31 (19–71)
Han JJ^136^	OAML	2015	90	Doxycycline (100 mg bid) for 3 weeks	27%	CR 24(27%) SD 34(37%) PD 31(34%)	40.5 (8–85)
Ferreri AJ^135^	OAML	2012	34	Doxycycline 100 mg bid for 3 weeks	65%	CR 6 (18%) PR 16 (47%) SD 11 (32%) PD 1 (3%)	37 (15–62)
Govi S^139^	OAML	2010	11	Clarithromycin 500 mg bid for 6 months	45%	CR 2 (18%) PR 3 (27%) SD 3 (27%) PD 3 (27%)	25 (12–35)

R: rituximab, PPI: proton-pump inhibitors
